# High-Fat Diet/Low-Dose Streptozotocin-Induced Type 2 Diabetes in Rats Impacts Osteogenesis and Wnt Signaling in Bone Marrow Stromal Cells

**DOI:** 10.1371/journal.pone.0136390

**Published:** 2015-08-21

**Authors:** Chao Qian, Chenyuan Zhu, Weiqiang Yu, Xinquan Jiang, Fuqiang Zhang

**Affiliations:** Department of Prosthodontics, School of Stomatology, Ninth People’s Hospital, Shanghai Jiao Tong University School of Medicine, Shanghai Key Laboratory of Stomatology. Shanghai, 200011, People’s Republic of China; Université de Lyon—Université Jean Monnet, FRANCE

## Abstract

Bone regeneration disorders are a significant problem in patients with type 2 diabetes mellitus. Bone marrow stromal cells (BMSCs) are recognized as ideal seed cells for tissue engineering because they can stimulate osteogenesis during bone regeneration. Therefore, the aim of this study was to investigate the osteogenic potential of BMSCs derived from type 2 diabetic rats and the pathogenic characteristics of dysfunctional BMSCs that affect osteogenesis. BMSCs were isolated from normal and high-fat diet+streptozotocin-induced type 2 diabetic rats. Cell metabolic activity, alkaline phosphatase (ALP) activity, mineralization and osteogenic gene expression were reduced in the type 2 diabetic rat BMSCs. The expression levels of Wnt signaling genes, such as β-catenin, cyclin D1 and c-myc, were also significantly decreased in the type 2 diabetic rat BMSCs, but the expression of GSK3β remained unchanged. The derived BMSCs were cultured on calcium phosphate cement (CPC) scaffolds and placed subcutaneously into nude mice for eight weeks; they were detected at a low level in newly formed bone. The osteogenic potential of the type 2 diabetic rat BMSCs was not impaired by the culture environment, but it was impaired by inhibition of the Wnt signaling pathway, likely due to an insufficient accumulation of β-catenin rather than because of GSK3β stimulation. Using BMSCs derived from diabetic subjects could offer an alternative method of regenerating bone together with the use of supplementary growth factors to stimulate the Wnt signaling pathway.

## Introduction

Diabetes mellitus (DM) is a pandemic metabolic disease that is characterized by an abnormal regulation of glucose metabolism and is associated with disorders such as cardiovascular disease, retinopathy, nephropathy, osteoporosis and impaired bone healing [1,2]. DM results from either an absolute deficiency of insulin (type 1 diabetes, T1DM) or from insulin resistance with/without abnormal insulin secretion (type 2 diabetes, T2DM). Out of all patients with DM, approximately 90–95% suffer from T2DM [3]. Recently, certain qualitative studies have reported that T1DM is associated with a decrease in bone mineral density (BMD), whereas in T2DM patients, BMD either remains unchanged or is slightly increased. However, it is widely accepted that T2DM carries a high risk of bone fracture and impaired bone healing, and disorders of the skeletal system might seriously lower a patient’s quality of life [4–7]. Although an increasing number of researches have been conducted on bone disorders caused by T2DM, little progress has been made in understanding the detailed pathogenesis of these bone disorders and/or effective treatment options for them [8,9].

In recent years, bone marrow stromal cells (BMSCs) have been investigated for use in the acceleration of wound healing following reconstructive and restorative surgeries [10]. BMSCs, which are a major source of osteoblasts, are a vital component of bone healing and regeneration. Recent studies have revealed that BMSCs can not only accelerate bone regeneration and healing through their effects on cell metabolism, proliferation and osteogenic differentiation in vitro but can also promote new bone formation in vivo. Therefore, BMSCs are considered an ideal cell source for tissue engineering [11,12]. Several studies have suggested that many types of cells, including epidermal stem cells and bone progenitor stem cells, might impair differentiation and function under diabetic conditions [13–15]. BMSCs have been autologously transplanted to treat diabetes mellitus by successfully restoring injured organs [16]; however, few studies have reported the use of BMSCs for bone repair in diabetes mellitus [17]. In a previous study, we found that the osteogenic differentiation of BMSCs was impaired in type 1 diabetics [18]. However, whether the osteogenic ability of BMSCs from type 1 diabetics is altered and what factors might alter this ability at the cellular and molecular levels are unknown.

The Wnt/β-catenin signaling pathway plays a central role in regulating cell growth and osteogenesis [19,20]. This pathway is stimulated by the accumulation of β-catenin and the inactivation of glycogen synthase kinase 3β (GSK3β) [21]. After translocation into the nucleus, β-catenin regulates the expression of runt-related transcription factor 2 (Runx2) and osterix (OSX), thereby strongly stimulating osteogenesis and new bone formation [22,23]. In addition, cyclin D1, which is a downstream gene target of β-catenin, is a main factor in the regulation of metabolism, proliferation and differentiation of cells [22,24]. Wnt signaling pathway activation promotes bone regeneration [25,26]. Zhong et al. investigated the expression levels of β-catenin and cyclin D1 in epidermal stem cells in diabetic rats. Their findings suggested that the inhibition of Wnt signaling might be an important mechanism behind delayed wound healing in individuals with DM [14]. However, whether the disruption of the Wnt/β-catenin pathway can alter the osteogenic abilities of BMSCs derived from type 2 diabetic rats has not yet been determined.

In the current study, the objective was to investigate and compare the metabolism, osteogenic differentiation and new bone formation abilities of BMSCs derived from type 2 diabetic rats with those derived from normal rats. The study also determined whether there were major changes in the Wnt signaling pathway in BMSCs derived from type 2 diabetic rats. A systematic evaluation of the osteogenic abilities of type 2 diabetic rat BMSCs could provide a basis for the pathogenesis underlying bone disorders in individuals with T2DM and a possible method of cell therapy to correct the associated bone defects.

## Materials and Methods

### Animal Care and Induction of a Type 2 Diabetic Rat Model

All animal experiments were approved by the Animal Research Committee of the Ninth People’s Hospital affiliated with the Shanghai Jiao Tong University School of Medicine. Twenty male Sprague Dawley rats (age: 8 weeks; body weight: 200–250 g) were housed in standard polypropylene cages (three rats/cage) under a 12-hour/12-hour light/dark cycle and an ambient temperature of 22–25°C. The rats were randomly divided into two groups: a normal group and a diabetic group. Type 2 diabetes was induced according to the method of Zhang et al. and Liu et al. [27,28]. The rats in the normal group were fed a regular chow diet consisting of a total kcal value of 20 kJ/kg (5% fat, 52% carbohydrate, 20% protein), whereas the rats in the diabetic group were placed on a high-fat diet with a total kcal value of 40 kJ/kg (20% fat, 45% carbohydrate, 22% protein). Both groups were maintained on their diets for eight weeks. During the fourth week, the rats in the diabetic group were treated with streptozotocin (STZ; Sigma-Aldrich, St Louis, MO). A single low dose of STZ (30 mg/kg, dissolved in 0.1 M sodium citrate buffer at pH 4.4) was injected into each rat intraperitoneally. After one week, blood glucose was tested using a blood glucose meter (Accu-Chek Performa; Roche Diagnostics, USA). Rats with blood glucose levels lower than 16.7 mmol/l were injected with STZ (30 mg/kg) a second time. The rats in the normal group were injected with a vehicle citrate buffer (0.25 ml/kg) at the same time. The above-described diets were maintained post-injection. At four weeks post injection, all rats with blood glucose concentrations greater than 16.7 mmol/l were considered to be diabetic and were selected for further research. The blood glucose concentrations and body weights of the rats were measured every week before and after injection. Blood insulin concentrations were obtained on the same day of injection and again during the fourth week after injection using an Iodine ^125^I Insulin Radioimmunoassay Kit.

### Isolation and Culture of Primary BMSCs from Normal and Diabetic Rats

The rats in the study were euthanized by cervical dislocation during the fourth week after injection. Their BMSCs were obtained by flushing the bone marrow from their tibias and femur bones using low-glucose Dulbecco’s modified Eagle’s medium (DMEM; Gibco BRL, Grand Island, USA) containing 10% fetal bovine serum (FBS; Gibco BRL, USA), 100 U/ml streptomycin, 100 U/ml penicillin and 200 U/ml heparin (Sigma-Aldrich, St Louis, MO), according to previously described procedures [29,30]. The primary cells were cultured in low-glucose DMEM supplemented with 10% FBS, 100 U/ml streptomycin and 100 U/ml penicillin at 37°C in a 5% CO_2_ atmosphere. Non-adherent cells were discarded via a change of medium after 24 hours, and the medium was replaced every three days until the cells reached 80–90% confluence (S1 Fig). A Z2 Coulter particle count and size analyzer (Beckman Coulter, USA) was used to evaluate the quantity of cells. The quantities of both normal and diabetic BMSCs were approximately 1.2–1.5×10^7^ cells/dish. Next, 0.25% trypsin (EDTA) was used for cell detachment, and the BMSCs were subcultured at a density of 1×10^5^ cells/ml. Cells at passage 2 or 3 were used for the subsequent experiments. Osteogenic medium (DMEM, 10% FBS, 10 mM dexamethasone, 50 μg/ml L-2-ascorbic acid and 10 mM glycerophosphate) was used to induce the osteogenic differentiation of BMSCs.

### Cell Metabolic Activity Analysis

As described in a previous study, an MTT assay was performed to investigate the metabolic activities of BMSCs from normal and type 2 diabetic rats at 1, 3, 5 and 7 days after culture in low-glucose DMEM (each group, n = 3) [31]. In brief, MTT solution (5 mg/ml) was added to each well in a 96-well plate, in which the cells had been plated at a density of 5×10^3^ cells/well. After incubation for four hours, the medium was extracted, and dimethyl sulfoxide (DMSO) was applied to dissolve the resultant formazan crystals. The absorbance was measured at 490 nm using an ELX Ultra Microplate Reader (Bio-Tek, VT, USA).

### Alkaline Phosphatase Staining and Activity Assay

To evaluate alkaline phosphatase (ALP) staining and activity, BMSCs were cultured in osteogenic medium for 7 days and 14 days. For ALP staining, the cells were treated with BCIP/NBT solution (Beyotime, China) in a dark environment after fixation for 15 min at 4°C. Regions that were positive for ALP staining displayed a purple color. The ALP activity was determined using p-nitrophenyl phosphate (Sigma-Aldrich, USA), as described in a previous study, while the cells were suspended in lysis buffer containing 2% NP-40 [32]. The absorbance was measured at 405 nm. The total protein content was determined using a Bio-Rad protein assay kit (Bio-Rad, USA) and measuring the absorbance at 630 nm and calculating the concentration according to a standard BSA (Sigma-Aldrich, USA) curve. ALP activity was indicated by the OD value at 405 nm, which was normalized to the total cellular protein. All experiments were performed in triplicate.

### Alizarin Red Staining and Mineralization Assay

BMSCs were cultured in osteogenic medium for 21 days before being subjected to alizarin red staining and calcium deposition assays. The cells were fixed with 95% ethanol and stained with 0.1% alizarin red S (Sigma-Aldrich, USA) for 30 minutes [33]. To quantify the mineralization, the stained cells were incubated with 100 mM cetylpyridinium chloride (Sigma-Aldrich, USA) for 1 h to release the calcium-bound stains into solution [34]. The absorbance of alizarin red S was determined at 590 nm. The results of the calcium deposition quantity assay were normalized and calculated as OD values per mg of total protein (n = 3 for each group).

### RNA Extraction and Real-Time Quantitative PCR Analysis

BMSCs were cultured for 7 days in osteogenic medium to detect gene expression related to osteogenesis or in low-glucose DMEM to detect gene expression related to the Wnt signaling pathway; total RNA was extracted using TRIzol reagent (Invitrogen, Carlsbad, USA). cDNA was acquired using a PrimeScript 1st strand cDNA synthesis kit (TaKaRa, Shiga, Japan). Four osteogenesis-related genes (alkaline phosphatase (ALP), osteocalcin (OCN), OSX, and Runx2) and four Wnt signaling pathway-related genes (β-catenin, GSK3β, cyclin D1 and myelocytomatosis oncogene (c-myc)) were assessed using a Bio-Rad real-time PCR system (Bio-Rad, USA). The relative expression levels of the genes were normalized to GAPDH, a housekeeping gene, according to the ΔΔCt method. The primers corresponding to the analyzed genes are listed in Table 1. All analyses were carried out in triplicate.

**Table 1:** Primer sequences for real-time polymerase chain reaction.

Gene	Primer sequence (F: forward; R: reverse)	Accession number
ALP	F: CACGTTGACTGTGGTTACTGCTGA	NM_013059.1
	R: CCTTGTAACCAGGCCCGTTG	
OCN	F: AAAGCCCAGCGACTCT	NM_013414.1
	R: CTAAACGGTGGTGCCATAGAT	
Osterix	F: CTATGCCAATGACTACCCACCC	NM_001037632.1
	R: CTGCCCACCACCTAACCAA	
Runx2	F: ACAACCACAGAACCACAAG	NM_001278483.1
	R: TCTCGGTGGCTGGTAGTGA	
β-catenin	F: AAGGTGCTGTCTGTCTGCTCTA	NM_053357.2
	R: CTTCCATCCCTTCCTGCTTAGT	
GSK-3β	F: ACCATCCTTATCCCTCCTCAC	NM_032080.1
	R: TTATTGGTCTGTCCACGGTCT	
Cyclin D1	F: GCGTACCCTGACACCAATCT	NM_171992.4
	R: GCTCCAGAGACAAGAAACGG	
c-myc	F: AAGAACAAGATGATGAGGAAG	NM_012603.2
	R: GTGCTGGTGAGTAGAGAC	
GAPDH	F: GGCACAGTCAAGGCTGAGAATG	NM_017008.4
	R: ATGGTGGTGAAGACGCCAGTA	

### Expression Analysis of Proteins Related to Osteogenesis and Wnt Signaling

Total protein was extracted from BMSCs using a mammalian cell extraction kit according to the manufacturer’s instructions (Biovision, Mountain View, CA). Aliquots of twenty micrograms of protein were separated via SDS-polyacrylamide gel electrophoresis and transferred to nitrocellulose membranes. The membranes were with Tris-buffered saline containing 5% skim milk and 0.05% Tween-20 for 2 hours at 37°C and then incubated at 4°C overnight with 1:1000 dilutions of the following primary antibodies: anti-β-catenin, anti-phosphorylated β-catenin (p-β-catenin), anti-GSK3β, anti-phosphorylated GSK3β (p-GSK3β), anti-cyclin D1, anti-c-myc, and anti-Runx2. In the case of the anti-GAPDH antibody, a 1:10,000 dilution was used. All of the antibodies were obtained from Cell Signaling Technology, USA. To remove unbound antibodies, the membranes were rinsed three times with Tris-buffered saline for 10 minutes each before being incubated in Tris-buffered saline containing a secondary antibody at a 1:5000 dilution for 1 hour at room temperature. Finally, a chemiluminescence reagent (Thermo, Waltham, MA) was applied to the membranes. The blots were exposed to Kodak X-ray film, and the grayscale values of each protein band were determined using NIH ImageJ 1.34. All of the results were normalized against GAPDH protein levels. The assays were conducted in triplicate.

### Surgical Implantation of Scaffolds with BMSCs

Porous calcium phosphate cement scaffolds (CPCs; Rebone, China) that were 4 mm in diameter and 2 mm in height were used in this study. The scaffolds were manufactured to have average pore diameters of 400 μm and 70% porosity. A 20-μl aliquot of low-glucose DMEM containing either normal or diabetic rat BMSCs was placed on each CPC scaffold at a density of 2×10^7^ cells/ml. The scaffolds were subcutaneously implanted into the backs of eight athymic nude mice obtained from the Ninth People’s Hospital Animal Center (Shanghai, China); each implant was placed near the center of the spine. The implants were placed vertically at 10-mm intervals. Each mouse received three different implants randomly placed in the back area from the following groups: the CPC scaffold group (n = 8), the normal BMSCs/CPC complex group (n = 8) or the diabetic BMSCs/CPC complex group (n = 8).

### Histological and Immunohistochemical Analyses

Histological and immunohistochemical analyses were performed on samples taken from the scaffold-implanted mice at 8 weeks post-implantation. Eight mice were euthanized by cervical dislocation, and their implants were extracted, fixed, decalcified and embedded with paraffin. The samples were then cut into 5-μm-thick sections and stained with hematoxylin and eosin (HE staining). Images were captured using a light microscope (Olympus, Japan). Areas exhibiting red staining with non-specific eosin staining around scaffold pore borders represented newly developed bone and/or osteoid. The areas were analyzed using an automated image analysis system (Image Pro 7.0; Media Cybernetics, USA). Three parallel sections were randomly selected from the serial sections of each sample and were measured to calculate the percentages of red-stained regions (new bone and osteoid area). These percentages were also calculated for the entire implant. New bone formation was quantified based on a ratio of the red-stained region to the entire implant.

Immunohistochemical analysis was conducted according to a previous study [18]. In brief, paraffin tissue slides were de-waxed, rehydrated and immersed in H_2_O_2_ for peroxidase quenching. Next, after blocking, the slides were incubated with a primary antibody against osteocalcin (OCN) (Santa Cruz Biotechnology, CA, USA) at 4°C overnight. Finally, an HRP-conjugated secondary antibody was added to the immersed slides, which were then maintained at room temperature for approximately one hour. Next, the color-developing reagent from a diaminobenzidine kit (dAB; Beyotime, China) was applied, and the slides were counterstained with hematoxylin. Photomicrographs were acquired via a light microscope (Olympus, Japan).

### Statistical Analysis

The data are expressed as the means ± standard deviation. Statistical analyses of the results were performed using Student’s independent samples t-test with SPSS v18.0 software (SPSS, Chicago, IL, USA). Differences were considered statistically significant at p<0.05.

## Results

### Experimental Rat Model of Type 2 Diabetes Mellitus

**Fig 1:**
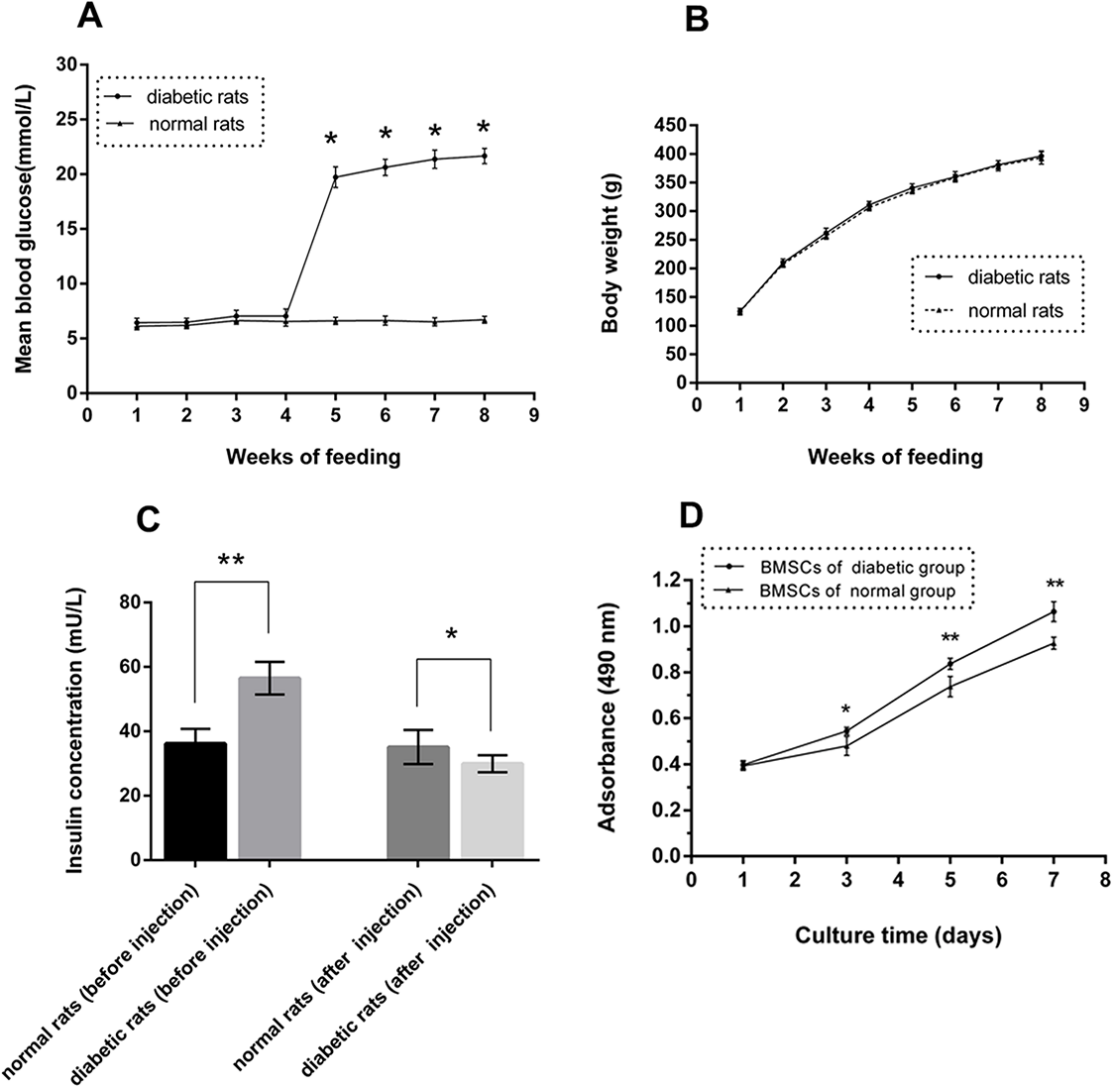
Changes in blood glucose levels, body weights and insulin concentrations in type 2 diabetic and normal rats during the experimental period and cell metabolic activity assay results. (A) Blood glucose was measured each week before and after injection (8 weeks). (B) Bodyweight was measured on the same day of every week (8 weeks). (C) Insulin concentration was measured at the four-week point prior to injection and again four weeks after injection (n = 10, *p<0.05, **p<0.01 versus normal rats). (D) Metabolic activity levels of BSMCs derived from type 2 diabetic and normal rats were analyzed on days 1, 3, 5, and 7 after culture in low-glucose DMEM via an MTT assay (n = 3, *p<0.05, **p<0.01 versus normal BMSCs).

The diabetic group of rats, which were treated with a four-week high-fat diet and multiple injections of low-dose STZ to induce diabetes, exhibited characteristic symptoms of diabetes mellitus, i.e., increased intake of food, polyuria, and polydipsia. The body weights of the diabetic group rats at four weeks before STZ injection were slightly higher than those of the normal group with no significant difference, and there were similar blood glucose levels between the groups. After STZ injection, the blood glucose levels of the diabetic group were significantly higher than those of the normal group. The body weights of the two groups were similar (Fig 1A and 1B, S1 Table). Compared with the normal group, the diabetic group exhibited significantly upregulated serum insulin concentrations prior to injection and significantly decreased serum insulin concentrations at four weeks post-injection (Fig 1C, S1 Table, p<0.05).

### Cell Metabolic Activity

The results from the cell metabolic activity analysis are shown in Fig 1D. On day 1, no significant difference was observed between the two groups. However, the metabolic activity levels of BMSCs from the type 2 diabetic rats were significantly decreased compared with those from the normal rats at days 3, 5, and 7 in low-glucose DMEM (p<0.05, S2 Table).

### ALP Activity and Calcium Deposition

**Fig 2:**
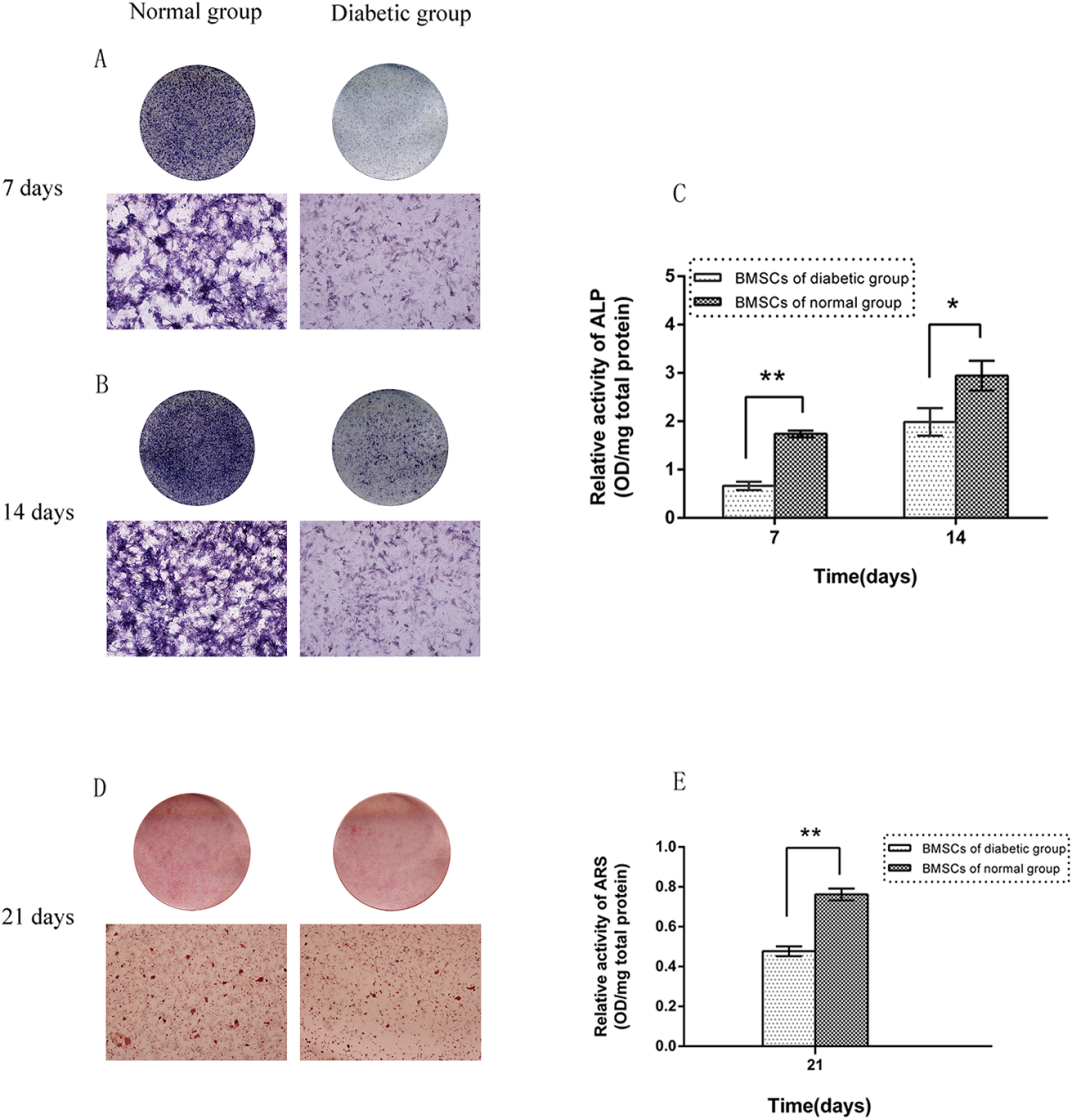
ALP activity and mineralization assays. (A, B) ALP staining of BMSCs derived from type 2 diabetic and normal rats on day 7 and day 14 of culture in osteogenic medium (lower images ×50). (C) ALP activity in BMSCs derived from type 2 diabetic and normal rats measured via a pNPP assay on day 7 and day 14. (D, E) Alizarin red S staining and quantitative mineralization assay of BMSCs derived from type 2 diabetic and normal rats on day 21 of culture in osteogenic medium (lower image of D ×50) (n = 3 for each group, *p<0.05, **p<0.01 versus normal BMSCs).

After 7 and 14 days in osteogenic medium, BMSCs from the type 2 diabetic rats displayed less pronounced ALP-positive areas than BMSCs from the normal rats (Fig 2A and 2B). Similarly, the quantification examination results showed that ALP activity in the diabetic rat BMSCs was significantly diminished by 38.3% at day 7 and by 67.5% at day 14 compared with that in normal rat BMSCs (p<0.05, Fig 2C). Furthermore, the results from alizarin red S staining and the quantitative assessment of calcium deposition revealed significantly reduced calcium deposition in the type 2 diabetic rat BMSCs compared with that in the normal rat BMSCs (p<0.05; Fig 2D and 2E; S2 Table).

### Expression Analysis of mRNAs Related to Osteogenesis and Wnt Signaling

**Fig 3:**
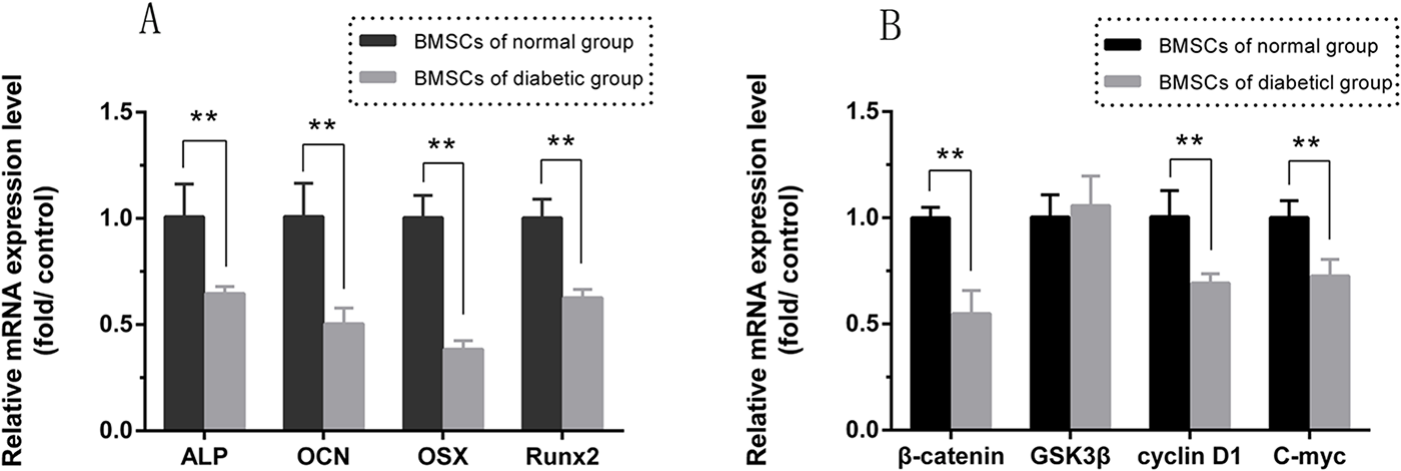
Gene expression levels of osteogenic differentiation and Wnt signaling markers. (A) Expression of the osteogenic differentiation-related genes ALP, OCN, OSX and Runx2 in BMSCs derived from type 2 diabetic and normal rats assayed at day 7 by real-time PCR. (B) The Wnt signaling markers β-catenin, GSK3β, cyclin D1 and c-myc measured at day 7 by real-time PCR (n = 3 for each group, **p<0.01 versus normal BMSCs).

Real-time quantitative PCR analysis of markers for osteogenic differentiation (ALP, OCN, OSX and Runx2) was performed using BMSCs that were cultured in osteogenic medium for 7 days (Fig 3A, S3 Table). An analysis of markers related to the Wnt signaling pathway (β-catenin, GSK3β, cyclin D1 and c-myc) was also performed using BMSCs that were incubated in low-glucose DMEM for 7 days (Fig 3B, S3 Table). Compared with the control (BMSCs from normal rats), the mRNA expression levels of ALP, OCN, OSX and Runx2 were significantly (p<0.01) decreased by 64.6%, 50.4%, 38.4% and 62.7%, respectively, in BMSCs derived from diabetic rats. Furthermore, the expression levels of β-catenin, cyclin D1 and c-myc were also significantly reduced in the type 2 diabetic rat BMSCs compared with the normal rat BMSCs (p<0.01), but the expression of GSK3β was unchanged.

### Expression Analysis of Proteins Related to Osteogenesis and Wnt Signaling

**Fig 4:**
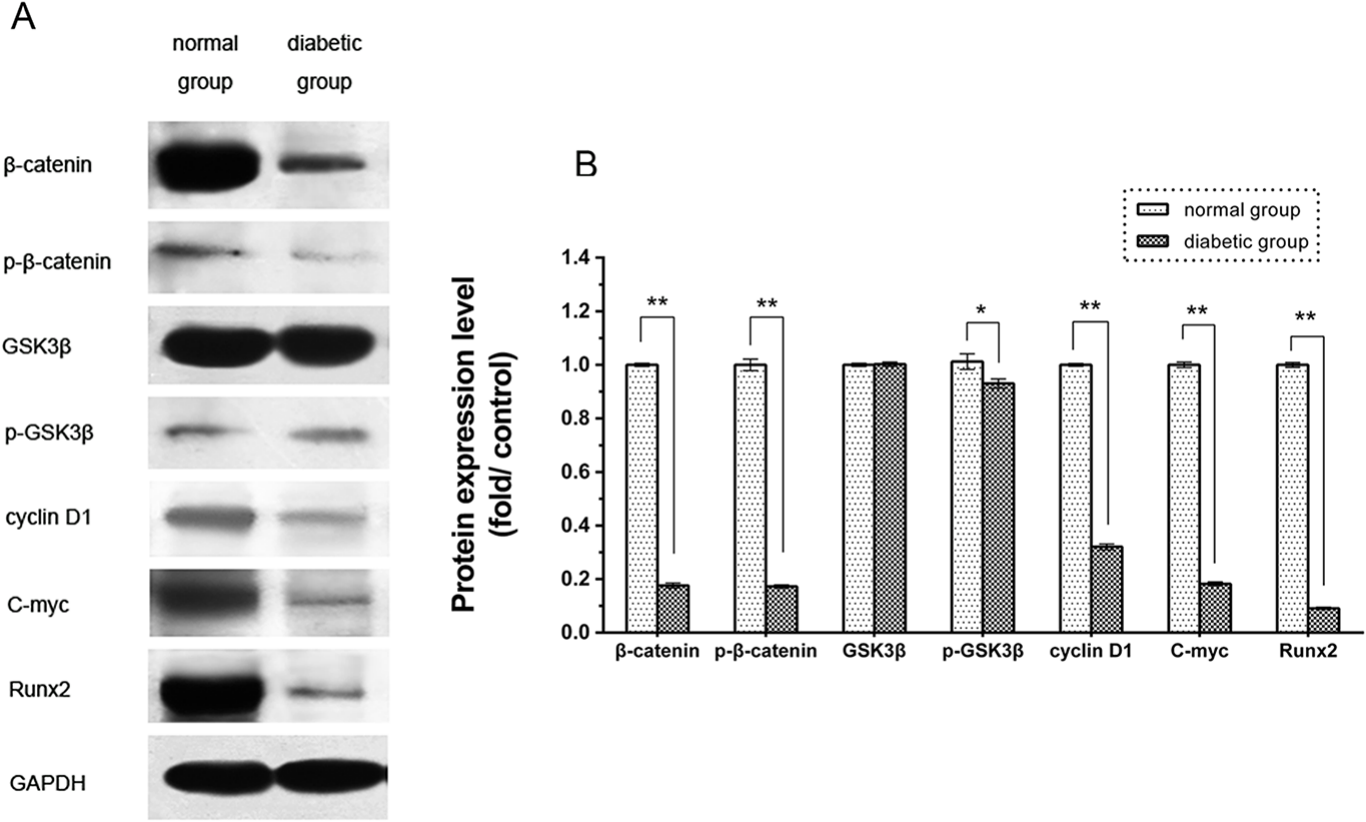
Western blot analysis of Wnt signaling pathway components. (A) Western blotting of β-catenin, p-β-catenin, GSK3β, p-GSK3β, cyclin D1, c-myc and Runx2. One representative image from three independently conducted assays is shown. (B) Protein levels of β-catenin, p-β-catenin, GSK3β, p-GSK3β, cyclin D1, c-myc and Runx2 quantified by densitometry (n = 3, *p<0.05, **p<0.01 versus normal BMSCs).

To evaluate the expression levels of proteins related to osteogenesis and Wnt signaling, BMSCs from both groups were cultured in low-glucose DMEM for seven days. The results are illustrated in Fig 4. A considerable decrease was observed in the protein expression levels of Runx2, β-catenin, p-β-catenin, cyclin D1 and c-myc in BMSCs from type 2 diabetic rats compared with BMSCs from normal rats (p<0.05, S3 Table). Nevertheless, the level of GSK3β protein expression in the diabetic rat BMSCs was slightly higher than that in the normal rat BMSCs, although the difference was not significant (p>0.05, S3 Table), and the level of p-GSK3β in the diabetic group was reduced compared with that in the normal group (p<0.05, S3 Table).

### Osteogenic Potential of Type 2 Diabetic and Normal Rat BMSCs In Vivo

**Fig 5:**
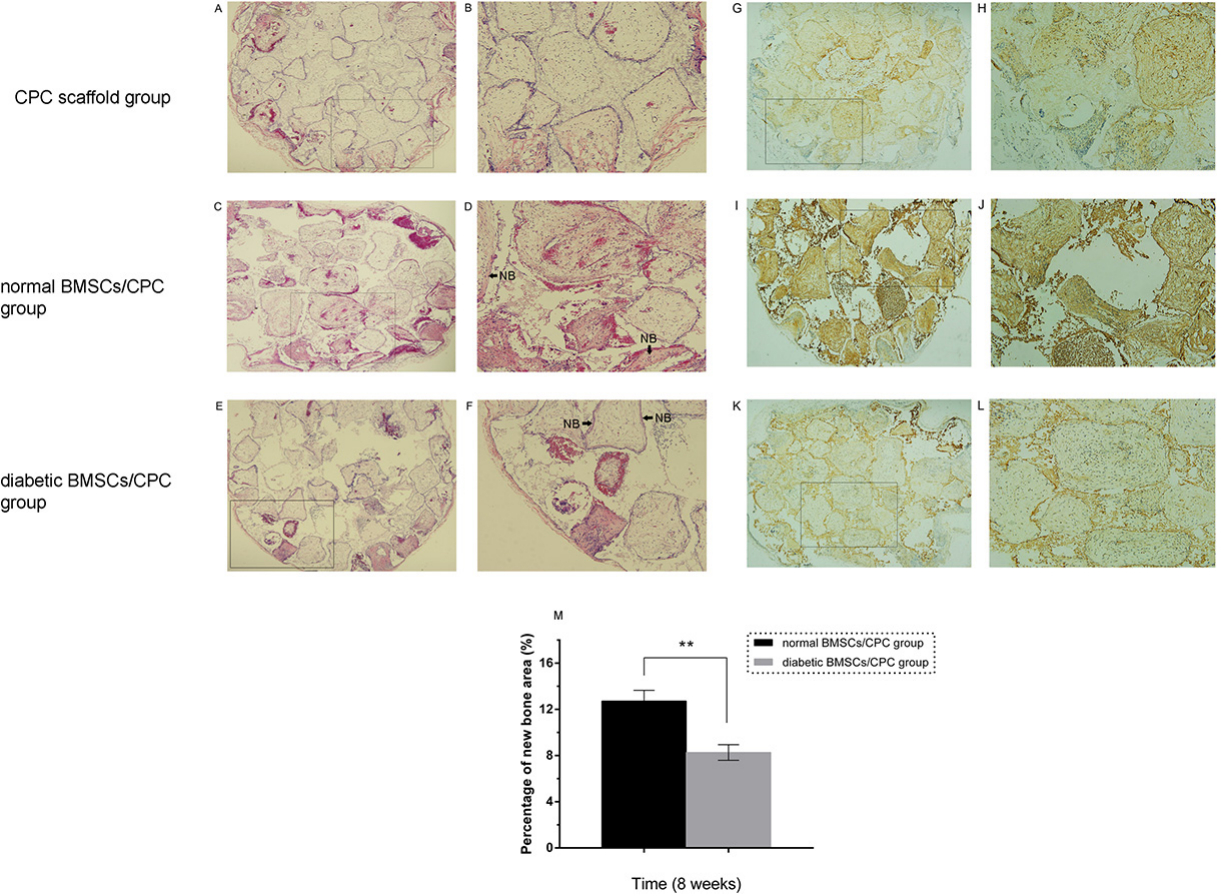
Histological and immunohistochemical findings at eight weeks. The red area around the pore of the scaffold represents new bone and osteoid area. Newly formed bone and osteoid was found in the type 2 diabetic rat BMSCs/CPC (E, F) and the normal rat BMSCs/CPC groups (C, D), but no new bone formation was found in the CPC scaffold group (A, B). NB: new bone and osteoid. Immunohistochemical staining of the OCN protein in the CPC scaffold group (G, H), type 2 diabetic rat BMSCs/CPC group (K, L), and normal rat BMSCs/CPC group (I, J). Percentages of new bone and osteoid area in the type 2 diabetic rat BMSCs/CPC and normal rat BMSCs/CPC groups were assessed via histomorphometric analysis (M) (A, C, E, G, I, K ×40; B, D, F, H, J, L ×100; n = 8, **p<0.01 versus normal rat BMSCs/CPC group).

BMSCs from both groups were cultured on CPC scaffolds and implanted into nude mice to further confirm the decreased osteogenic potential of type 2 diabetic rat BMSCs in vitro. At the eighth week after implantation, less new bone and osteoid formation was observed in the diabetic rat BMSCs/CPC complex group than in the normal rat BMSCs/CPC complex group (Fig 5C–5F). No newly formed bone was detected in the CPC scaffold group (Fig 5A and 5B). Histomorphometric analysis showed that the percentage of new bone and osteoid area in the diabetic rat BMSCs/CPC complex group (8.27±0.30%) was significantly less (65% of the normal value; p<0.05) than that in the normal rat BMSCs/CPC complex group (12.73±0.41%) (Fig 5M, S4 Table). In addition, immunohistochemical staining of OCN in the diabetic group was less intense compared with that in the normal group (Fig 5G–5L). These in vivo results further supported our in vitro data demonstrating that type 2 diabetes mellitus impairs the osteogenic potential of BMSCs.

## Discussion

Diabetes mellitus is a metabolic disease that can result in a series of disorders of the skeletal system. Even with normal BMD, T2DM patients still face a high risk of bone fractures and impaired bone healing. Recent researches have shown that these disorders primarily result from impaired bone formation rather than from bone resorption [2,35,36]. Previous studies have concentrated on the abnormal osteogenesis of skeletal tissues in DM and on the depressed proliferation, osteogenic differentiation and new bone formation ability of osteoblast cells in T1DM [5,37], but not on osteoblast cells in T2DM. Furthermore, BMSCs are considered an ideal cell for bone restoration in tissue engineering because they are a major component of bone formation and regeneration. In a previous study, we investigated BMSC osteogenesis in T1DM [18]. However, research on whether BMSC osteogenic ability in T2DM changes in a manner similar to that in T1DM has not yet been reported, and the related mechanisms of such changes remain unclear. In the current study, we confirmed that the osteogenic potential of BMSCs derived from type 2 diabetic rats was reduced relative to those derived from normal rats both in vitro and in vivo. Furthermore, we demonstrated that inhibition of the Wnt signaling pathway might also contribute to depressed osteogenic differentiation and impairment of new bone formation in BMSCs from type 2 diabetics.

A high-fat diet and multiple low doses of streptozotocin were used to develop an improved and more stable type 2 diabetic rat model from which to acquire BMSCs. According to many previous studies, feeding rats a high-fat diet can promote the development of insulin resistance [27,38]. Injections of high doses of STZ have been shown to critically damage pancreatic β-cell functioning, leading to insulin secretion, which is considered to resemble T1DM [38]. Recently, multiple low-dose injections of STZ have been reported to induce a gradual impairment of insulin secretion, which is similar to the natural progression of T2DM in humans [27,28]. Therefore, in the current study, a high-fat diet and multiple low-dose injections of STZ (30 mg/kg injected twice at weekly intervals) were adopted to induce type 2 diabetes in rats according to the methods of Zhang et al. and Srinivasan et al. [27,39]. Hyperglycemia and insulin resistance are the key symptoms of type 2 diabetes mellitus. The high blood glucose levels and low insulin concentrations of the induced rats are consistent with the features of T2DM. Thus, we successfully developed a stable type 2 diabetic rat model and were able to evaluate the osteogenic potential of BMSCs derived from these rats.

In the current study, the osteogenic potential of type 2 diabetic rat BMSCs in vitro was assayed based on cell metabolic activity, ALP activity and calcium deposition. The results showed that the cell metabolic activity in diabetic rat BMSCs significantly declined compared with that in normal rat BMSCs. The cell metabolic activity could to some extent represent the cell proliferation rate. Therefore, the results suggested that the metabolic activity and proliferation rate of BMSCs were depressed in the diabetic environment, an observation that is in agreement with previous studies [18,40]. The decreased ALP activity and reduced calcium deposition also demonstrated the decreased osteogenic potential of diabetic rat BMSCs in vitro. The decreased osteogenic ability of the type 2 diabetic rat BMSCs was similar to that reported in our previous study on BMSCs in T1DM [18], which might explain the relationship between DM and impaired bone regeneration. The osteogenic abilities of BMSCs derived from type 2 diabetic and normal rats were also compared at the mRNA level. Four osteogenic markers, ALP, OCN, OSX and Runx2, were selected for study. ALP is adjusted in response to phosphate metabolism and acts as a marker for early osteogenic cell differentiation [41]. OCN, which is a product of the deposition and mineralization of osteoblasts, is considered a marker for later stages of osteogenic differentiation [42]. OSX is recognized as a key gene in osteogenic maturation and is associated with the Wnt signaling pathway [43]. Runx2, an osteoblast transcription activator, is necessary for osteogenic differentiation. In the early stages of osteogenesis, Runx2 regulates the expression of osteogenic genes and is also expressed downstream of the Wnt signaling pathway [37,44]. The results of our real-time PCR analysis indicated that the mRNA expression levels of all of the evaluated osteogenic genes were decreased in BMSCs from type 2 diabetic rats compared with those from normal rats. The low expression levels of ALP, OCN, OSX and Runx2 suggested that the osteogenic potential of type 2 diabetic rat BMSCs was severely impaired. These results further suggested that BMSCs in T2DM patients could serve as potential targets for the treatment of bone disorders and that further study to improve their osteogenic ability is warranted.

Histological and immunohistochemical analyses were conducted to evaluate new bone and osteoid formation by CPC scaffold-associated type 2 diabetic rat BMSCs. A well-defined model of ectopic bone formation was used in the current study. BMSCs from diabetic rats were cultured on CPC scaffolds and implanted in vivo to further reveal their osteogenic potential, which was demonstrated to be impaired in vitro. In the nude mouse model that was employed, osteogenic differentiation and new bone and osteoid formation were detected in both the diabetic and normal BMSC groups. The overall area of new bone and osteoid in the diabetic group was smaller than that in the normal group (i.e., reduced by 65%) according to histological analysis. Additionally, immunohistochemical analysis indicated that diabetic rat BMSCs showed impaired osteogenic differentiation with low expression of the OCN protein. The results verified that the osteogenic capability of type 2 diabetic rat BMSCs was impaired both in vitro and in vivo, which is similar to our previous findings on type 1 diabetic rat BMSCs [18]. Because new bone was detected in the diabetic group, diabetic rat BMSCs might offer a possible source of stem cells for the treatment of diabetic bone disorders, although this prospect requires further investigation.

We cultured type 2 diabetic rat BMSCs in low-glucose DMEM to understand the mechanism driving their osteogenic potential. Studies have found that hyperglycemia plays an important role in bone disorders in diabetes mellitus [45]. Therefore, we selected low-glucose DMEM as a culture medium to avoid the effects caused by high-glucose DMEM on cell proliferation and differentiation. The results indicate that in a low-glucose culture environment, the impairments observed in cell proliferation and osteogenic differentiation in diabetic rat BMSCs might be permanent and not merely related to hyperglycemia. These findings are in agreement with those of our previous study [18].

The Wnt signaling pathway, which is a crucial signaling pathway in cell maturity and osteogenesis, was evaluated in the current study. Recent studies have indicated that it is an important signal transduction pathway in regulating cell growth, differentiation and tissue morphogenesis [20,22,23,46]. Genes related to Wnt signaling, including β-catenin, GSK3β, cyclin D1 and c-myc, were measured at both the mRNA and protein levels. β-catenin is a key factor of Wnt signaling and possesses multiple functions in the regulation of cell proliferation and differentiation. An accumulation of β-catenin will activate the Wnt signaling pathway [20,22,47]. GSK3β is commonly viewed as an inhibitor of Wnt signaling [20,25]. The activation of GSK3β leads to the degradation of β-catenin, which reduces the expression of β-catenin [26,48]. Moreover, GSK3β is a major factor in insulin resistance via its role in the regulation of the insulin signaling pathway [49,50]. Cyclin D1 and c-myc are the significant target genes of the Wnt signaling pathway and cause alterations to cell cycle, cell proliferation and cell survival [24,51]. Wnt signaling also regulates the expression of Runx2, an osteogenic transcription factor. The activation of the Wnt signaling pathway strongly accelerates osteogenesis [22,52]. In diabetic rats, Wnt signaling plays a central role in diabetic wound healing. It has been reported that the survival time for exogenous bone mesenchymal stem cells in diabetic wounds is short, which might be partially due to dysfunctional Wnt signaling [53]. In addition, the expression levels of β-catenin and GSK3β are significantly reduced in the femurs and tibias of type 1 diabetic rats [37]. Therefore, alterations in the Wnt signaling pathway might impact the osteogenic potential of type 2 diabetic rat BMSCs. In the current study, the major changes in Wnt signaling in type 2 diabetic rat BMSCs were decreased mRNA and protein expression levels of β-catenin, cyclin D1, c-myc and Runx2. The level of p-β-catenin was also significantly diminished, perhaps due to the low expression of β-catenin, leading to decreased phosphorylation of β-catenin. The decreased expression levels of cyclin D1 and c-myc might have affected the proliferation ability of the diabetic rat BMSCs. However, the expression of GSK3β was the same between BMSCs derived from diabetic versus normal rats, while the expression of p-GSK3β was downregulated in diabetic rat BMSCs. Previous studies have suggested that abnormal activation of GSK3β and decreased levels of p-GSK3β can depress glycogen synthesis and inhibit the insulin signaling pathway, which contributes to in the induction of insulin resistance in diabetes mellitus [49,50]. Despite the lack of changes in the expression level of GSK3β observed in the present study, the result of p-GSK3β expression level indicated that the activity of GSK3β was enhanced while the phosphorylation of GSK3β was decreased in BMSCs of type 2 diabetic rats, which might reflect the reported involvement of GSK3β in insulin resistance. These results suggested that the proliferation ability and osteogenic potential of type 2 diabetic rat BMSCs were impaired following the inhibition of Wnt signaling, likely because of an insufficient accumulation of β-catenin rather than as a response to GSK3β activation.

In conclusion, decreased osteogenic potential was observed in BMSCs derived from type 2 diabetic rats, which was not merely due to the cell culture environment. Their impaired osteogenic potential might be partially related to the inhibition of Wnt signaling as a result of insufficient β-catenin accumulation rather in response to GSK3β stimulation. These results may enhance our understanding of the bone disorders that are related to T2DM, and a key aspect of bone disorder treatment could be improving the osteogenic ability of BMSCs in T2DM patients. Moreover, BMSCs from individuals with T2DM may still offer an alternative for bone regeneration in tandem with the supplementation of certain growth factors to activate the Wnt signaling pathway.

## Supporting Information

S1 Fig The growth of normal and diabetic rat BMSCs. (TIF)

S2 Fig ALP and alizarin red S staining. (TIF)

S1 Table The blood glucose levels, body weights and insulin concentrations in type 2 diabetic and normal rats during the experimental period. (DOC)

S2 Table Cell metabolic activity, ALP activity and mineralization assays. (DOC)

S3 Table Gene expression levels of osteogenic differentiation and Wnt signaling markers and western blot analysis of Wnt signaling pathway components. (DOC)

S4 Table Percentages of new bone areas in the type 2 diabetic BMSCs/CPC group and the normal BMSCs/CPC group. (DOC)
