# Supplementary figures and images for: High-Fat Diet/Low-Dose Streptozotocin-Induced Type 2 Diabetes in Rats Impacts Osteogenesis and Wnt Signaling in Bone Marrow Stromal Cells
Source: PLoS One. 2015 Aug 21;10(8):e0136390. doi: 10.1371/journal.pone.0136390 (PMC4546646; doi:10.1371/journal.pone.0136390)

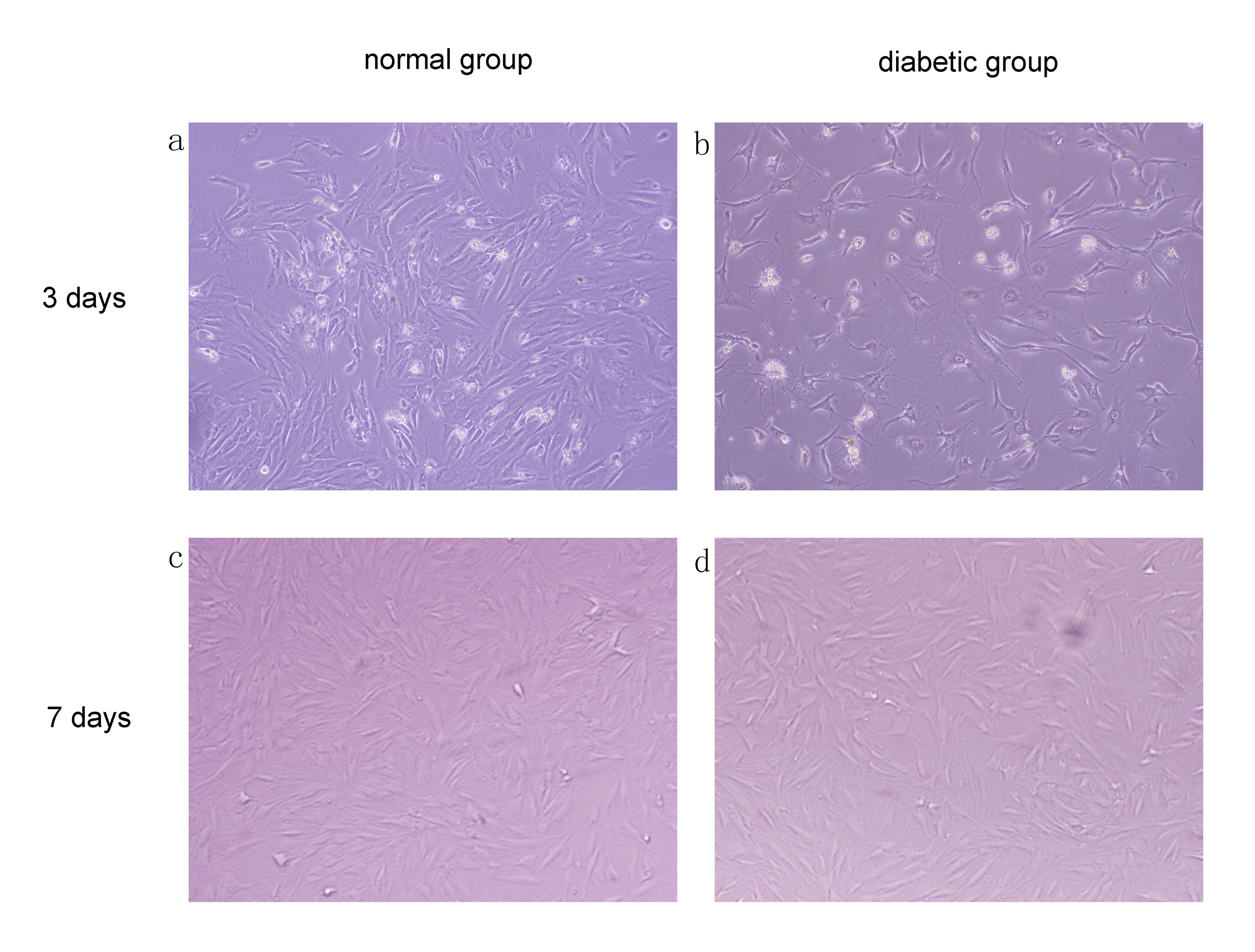

Supplement: S1 Fig — (TIF) [file pone.0136390.s001.tif]

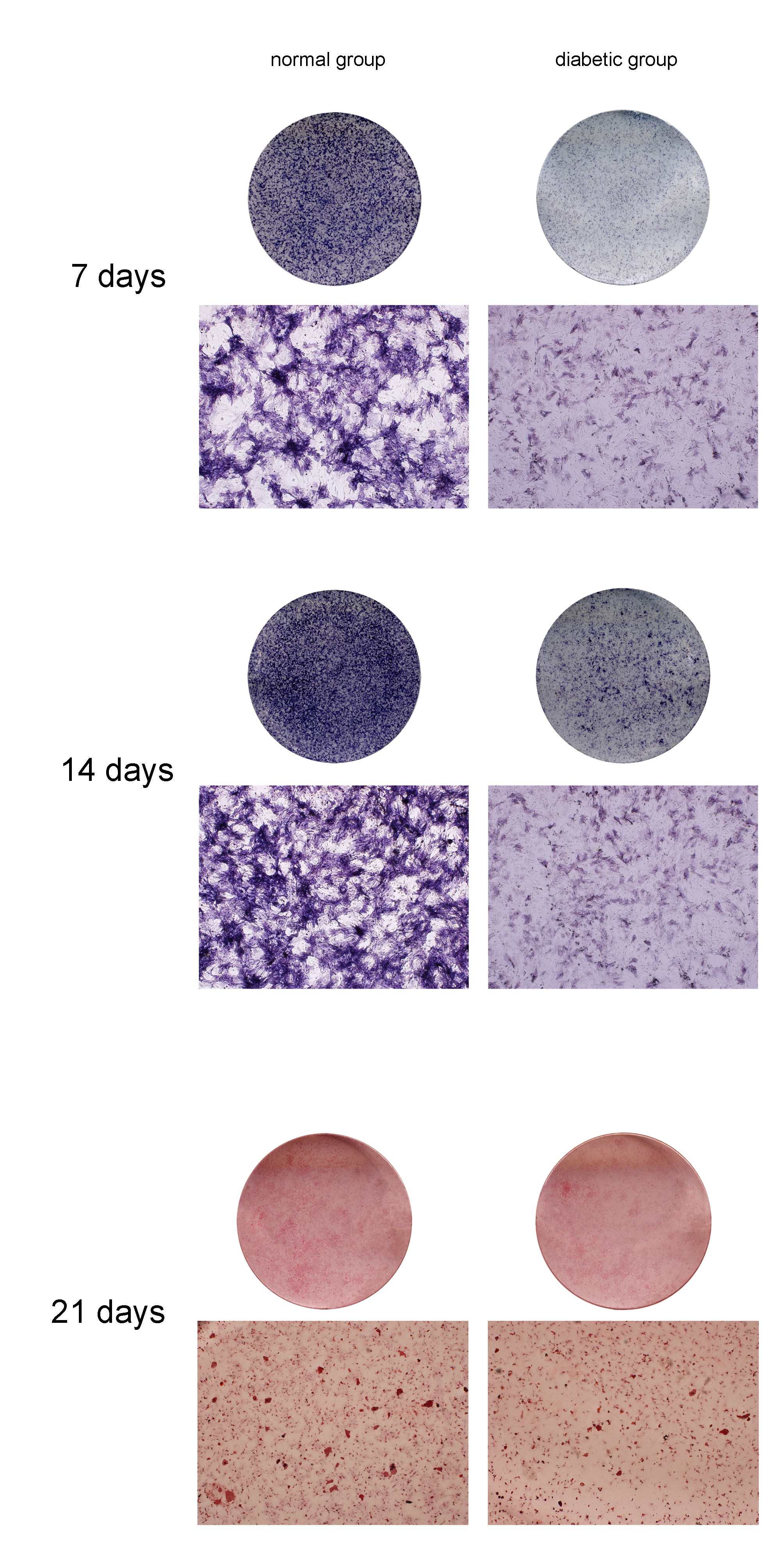

Supplement: S2 Fig — (TIF) [file pone.0136390.s002.tif]
