# Supplementary material for: High-Fat Diet/Low-Dose Streptozotocin-Induced Type 2 Diabetes in Rats Impacts Osteogenesis and Wnt Signaling in Bone Marrow Stromal Cells
Source: PLoS One. 2015 Aug 21;10(8):e0136390. doi: 10.1371/journal.pone.0136390 (PMC4546646; doi:10.1371/journal.pone.0136390)
Supplement: S1 Table — (DOC) [file pone.0136390.s003.doc]

Table S1 The data for blood glucose levels, body weights and insulin concentrations in type 2 diabetic and normal rats during the experimental period.

**Changes in blood glucose levels in type 2 diabetic and normal rats during the experimental period (mmol/L)**

| Weeks | diabetic rats | | | | | | | | | | normal rats | | | | | | | | | |
| --- | --- | --- | --- | --- | --- | --- | --- | --- | --- | --- | --- | --- | --- | --- | --- | --- | --- | --- | --- | --- |
| 1. | 6.3 | 6.5 | 6.8 | 5.9 | 6.3 | 6.7 | 5.8 | 6.5 | 7.1 | 6.6 | 6.5 | 5.8 | 5.7 | 6.1 | 5.7 | 6.4 | 5.9 | 6.6 | 6.2 | 6.4 |
| 2. | 6.9 | 6.7 | 6.3 | 6.4 | 7.0 | 6.5 | 5.7 | 6.3 | 6.5 | 6.6 | 6.2 | 6.3 | 5.9 | 6.6 | 6.0 | 6.5 | 5.8 | 6.4 | 5.8 | 6.5 |
| 3. | 6.4 | 7.5 | 6.9 | 6.4 | 7.1 | 7.3 | 7.8 | 7.5 | 7.3 | 6.2 | 6.5 | 6.9 | 6.8 | 6.7 | 6.1 | 7.1 | 6.3 | 6.4 | 6.5 | 7.0 |
| 4. | 7.6 | 7.2 | 6.9 | 7.5 | 7.2 | 7.5 | 7.1 | 5.8 | 7.7 | 6.0 | 6.2 | 6.5 | 6.8 | 6.4 | 6.8 | 6.7 | 6.5 | 7.2 | 6.9 | 5.6 |
| 5. | 19.7 | 19.5 | 20.2 | 19.6 | 19.9 | 19.3 | 20.5 | 21.6 | 18.9 | 18.1 | 6.7 | 6.5 | 6.2 | 7.0 | 6.5 | 6.8 | 6.8 | 7.1 | 6.1 | 6.4 |
| 6. | 20.4 | 19.6 | 20.5 | 22.1 | 21.3 | 21.2 | 19.8 | 20.7 | 20.5 | 20.2 | 6.9 | 6.4 | 6.0 | 6.3 | 6.6 | 7.1 | 6.2 | 7.0 | 7.2 | 6.7 |
| 7. | 21.2 | 21.5 | 21.4 | 22.3 | 22.5 | 21.8 | 21.6 | 21.5 | 20.1 | 19.9 | 7.1 | 6.5 | 6.9 | 5.9 | 6.2 | 6.8 | 6.3 | 6.7 | 6.2 | 6.6 |
| 8. | 21.3 | 22.7 | 22.1 | 21.9 | 22.2 | 20.8 | 21.5 | 20.5 | 21.4 | 22.3 | 6.8 | 7.1 | 6.9 | 6.1 | 6.9 | 6.7 | 6.6 | 6.7 | 6.3 | 7.0 |

**Changes in body weights in type 2 diabetic and normal rats during the experimental period (g)**

| Weeks | diabetic rats | | | | | | | | | | normal rats | | | | | | | | | |
| --- | --- | --- | --- | --- | --- | --- | --- | --- | --- | --- | --- | --- | --- | --- | --- | --- | --- | --- | --- | --- |
| 1. | 115. | 130. | 120. | 130. | 125. | 130. | 125. | 125. | 120. | 130. | 120. | 125. | 135. | 120. | 130. | 125. | 120. | 115. | 125. | 130. |
| 2. | 200. | 205. | 210. | 210. | 215. | 220. | 200. | 215. | 215. | 210. | 200. | 210. | 205. | 215. | 215. | 210. | 205. | 210. | 200. | 205. |
| 3. | 260. | 250. | 270. | 270. | 255. | 250. | 260. | 270. | 270. | 265. | 250. | 255. | 250. | 260. | 260. | 250. | 265. | 255. | 260. | 260. |
| 4. | 305. | 315. | 315. | 315. | 305. | 315. | 310. | 320. | 300. | 310. | 310. | 310. | 305. | 300. | 300. | 315. | 305. | 300. | 315. | 305. |
| 5. | 350. | 335. | 350. | 340. | 340. | 350. | 340. | 335. | 330. | 335. | 330. | 345. | 335. | 330. | 340. | 340. | 330. | 335. | 335. | 335. |
| 6. | 355. | 355. | 355. | 360. | 350. | 365. | 355. | 355. | 370. | 380. | 350. | 355. | 355. | 360. | 355. | 365. | 370. | 360. | 355. | 360. |
| 7. | 365. | 390. | 380. | 385. | 385. | 390. | 380. | 375. | 380. | 380. | 380. | 370. | 375. | 390. | 380. | 395. | 375. | 385. | 380. | 365. |
| 8. | 400. | 405. | 390. | 395. | 395. | 385. | 410. | 395. | 405. | 385. | 395. | 385. | 385. | 410. | 385. | 390. | 400. | 405. | 400. | 375. |

**Changes in insulin concentrations in type 2 diabetic and normal rats during the experimental period (mU/L)**

| normal rats (before injection) | diabetic rats (before injection) | normal rats (after injection) | diabetic rats (after injection) |
| --- | --- | --- | --- |
| 35.9 | 57.4 | 36.7 | 28.7 |
| 37.6 | 57.9 | 39.6 | 31.6 |
| 39.4 | 62.4 | 30.4 | 29.4 |
| 30.5 | 50.4 | 31.6 | 33.7 |
| 28.7 | 59.2 | 35.5 | 31.4 |
| 31.6 | 65.1 | 42.3 | 24.2 |
| 40.8 | 52.3 | 35.5 | 29.9 |
| 42.5 | 53.9 | 42.4 | 31.9 |
| 38.8 | 56.8 | 30.1 | 30.7 |
