# Supplementary material for: High-Fat Diet/Low-Dose Streptozotocin-Induced Type 2 Diabetes in Rats Impacts Osteogenesis and Wnt Signaling in Bone Marrow Stromal Cells
Source: PLoS One. 2015 Aug 21;10(8):e0136390. doi: 10.1371/journal.pone.0136390 (PMC4546646; doi:10.1371/journal.pone.0136390)
Supplement: S2 Table — (DOC) [file pone.0136390.s004.doc]

Table S2 The data for cell metabolic activity, ALP activity and mineralization assays.

**Cell metabolic activity assay**

| Culture time (days) | BMSCs of normal group | | | | | BMSCs of diabetic group | | | | |
| --- | --- | --- | --- | --- | --- | --- | --- | --- | --- | --- |
| 1. | 0.385 | 0.363 | 0.400 | 0.412 | 0.401 | 0.396 | 0.374 | 0.421 | 0.408 | 0.389 |
| 3. | 0.458 | 0.518 | 0.527 | 0.459 | 0.435 | 0.568 | 0.547 | 0.526 | 0.551 | 0.532 |
| 5. | 0.717 | 0.798 | 0.680 | 0.757 | 0.736 | 0.821 | 0.805 | 0.854 | 0.867 | 0.833 |
| 7. | 0.925 | 0.913 | 0.955 | 0.891 | 0.948 | 1.056 | 0.992 | 1.079 | 1.103 | 1.086 |

**ALP activity assays**

| BMSCs of normal group | | | BMSCs of diabetic group | | |
| --- | --- | --- | --- | --- | --- |
| 7day | 1.740766 | 1.804314 | 0.664996 | 0.575909 | 0.747109 |
| 14day | 2.592292 | 3.167553 | 2.241792 | 2.038892 | 1.679209 |

**Mineralization assays**

| BMSCs of diabetic group | | | BMSCs of normal group | | |
| --- | --- | --- | --- | --- | --- |
| 0.453 | 0.476 | 0.502 | 0.732 | 0.763 | 0.791 |
