# Supplementary material for: High-Fat Diet/Low-Dose Streptozotocin-Induced Type 2 Diabetes in Rats Impacts Osteogenesis and Wnt Signaling in Bone Marrow Stromal Cells
Source: PLoS One. 2015 Aug 21;10(8):e0136390. doi: 10.1371/journal.pone.0136390 (PMC4546646; doi:10.1371/journal.pone.0136390)
Supplement: S3 Table — (DOC) [file pone.0136390.s005.doc]

Table S3 Gene expression levels of osteogenic differentiation and Wnt signaling markers, and western blot analysis for the Wnt signaling pathway.

**Gene expression levels of osteogenic differentiation and Wnt signaling markers**

|  | BMSCs of normal group | | | | BMSCs of diabetic group | | | |
| --- | --- | --- | --- | --- | --- | --- | --- | --- |
| ALP | 1.029897 | 1.216301 | 0.915416 | 0.872060 | 0.688964 | 0.642826 | 0.608150 | 0.647297 |
| OCN | 1.162717 | 1.123111 | 0.899690 | 0.851159 | 0.523950 | 0.431520 | 0.465709 | 0.597703 |
| OSX | 0.869043 | 1.099997 | 1.069918 | 0.977725 | 0.437544 | 0.388908 | 0.343290 | 0.365388 |
| Runx2 | 0.952638 | 0.926588 | 1.125058 | 1.006956 | 0.578344 | 0.659754 | 0.615572 | 0.655197 |

|  | BMSCs of normal group | | | | BMSCs of diabeticl group | | | |
| --- | --- | --- | --- | --- | --- | --- | --- | --- |
| β-catenin | 1.069918 | 0.991373 | 0.984525 | 0.957603 | 0.446738 | 0.465709 | 0.627419 | 0.658612 |
| GSK3β | 1.160704 | 0.949342 | 0.942785 | 0.962594 | 1.053361 | 1.185093 | 1.128964 | 0.867539 |
| cyclin D1 | 1.127010 | 1.073632 | 0.974342 | 0.848214 | 0.713260 | 0.629597 | 0.708333 | 0.723217 |
| C-myc | 1.075494 | 1.017480 | 1.024557 | 0.891929 | 0.729510 | 0.724471 | 0.630689 | 0.820742 |

**Western blot analysis for the Wnt signaling pathway**

|  | normal group | | | diabetic group | | |
| --- | --- | --- | --- | --- | --- | --- |
| β-catenin | 1.000354 | 0.9934617 | 1.006184 | 0.1746726 | 0.1861656 | 0.1671847 |
| p-β-catenin | 0.998622 | 1.022106 | 0.979272 | 0.1779848 | 0.166574 | 0.1738647 |
| GSK3β | 0.9996515 | 0.9940252 | 1.006323 | 1.002892 | 0.9940361 | 1.010215 |
| p-GSK3β | 0.9997432 | 0.9913813 | 1.045992 | 1.205990 | 1.236302 | 1.239142 |
| cyclin D1 | 0.9979569 | 1.006145 | 0.9958985 | 0.3195286 | 0.3102221 | 0.331542 |
| C-myc | 0.9996628 | 1.010222 | 0.9901156 | 0.1793022 | 0.1768326 | 0.191059 |
| Runx2 | 1.000947 | 0.9904695 | 1.008583 | 0.09173865 | 0.08594117 | 0.09468647 |
