# Supplementary material for: High-Fat Diet/Low-Dose Streptozotocin-Induced Type 2 Diabetes in Rats Impacts Osteogenesis and Wnt Signaling in Bone Marrow Stromal Cells
Source: PLoS One. 2015 Aug 21;10(8):e0136390. doi: 10.1371/journal.pone.0136390 (PMC4546646; doi:10.1371/journal.pone.0136390)
Supplement: S4 Table — (DOC) [file pone.0136390.s006.doc]

Table S4 Percentages of new bone area in the type 2 diabetic BMSCs/CPC group and normal BMSCs/CPC group.

Percentages of new bone area in the type 2 diabetic BMSCs/CPC group and normal BMSCs/CPC group

|  | normal BMSCs/CPC group | | | | | diabetic BMSCs/CPC group | | | | |
| --- | --- | --- | --- | --- | --- | --- | --- | --- | --- | --- |
| Time (8 weeks) | 12.95 | 13.76 | 11.62 | 11.95 | 13.37 | 8.49 | 7.47 | 9.25 | 7.83 | 8.32 |
